# Supplementary material for: Discovery and application of insertion-deletion (INDEL) polymorphisms for QTL mapping of early life-history traits in Atlantic salmon
Source: BMC Genomics. 2010 Mar 8;11:156. doi: 10.1186/1471-2164-11-156 (PMC2838853; doi:10.1186/1471-2164-11-156)
Supplement: Additional file 2 — Information on developed 76 locus single-run INDEL panel in Atlantic salmon. Information on fluorescence labeling, primer concentrations, PCR pooling and links to alignments, INDEL motifs and GENESCAN (Burge and Karlin 1997) predictions of genes/exons are available in html format. [file 1471-2164-11-156-S2.ZIP › Additionalfile2/table9520.html]

Contig Alignment

```
Base	A B C D E F G H I J K L M N O P Q   
1	. C . C . . . . . . . . . . . . .    
2	. A . A . . . . . . . . . . . . .    
3	. G . G . . . . . . . . . . . . .    
4	. T . T . . . . . . . . . . . . .    
5	. T . T . . . . . . . . . . . . .    
6	. A . A . . . . . . . . . . . . .    
7	. C . C . . . . . . . . . . . . .    
8	. A . A . . . . . . . . . . . . .    
9	. C . C . . . . . . . . . . . . .    
10	. G . G . . . . . . . . . . . . .    
11	. A . A . . . . . . . . . . . . .    
12	. A . A . . . . . . . . . . . . .    
13	. T . T . . . . . . . . . . . . .    
14	. T . T . . . . . . . . . . . . .    
15	. T . T . . . . . . . . . . . . .    
16	. A . A . . . . . . . . . . . . .    
17	. T . T . . . . . . . . . . . . .    
18	. T . T . . . . . . . . . . . . .    
19	. A . A . . . . . . . . . . . . .    
20	. G . G . . . . . . . . . . . . .    
21	. G . G . . . . . . . . . . . . .    
22	. G . G . . . . . . . . . . . . .    
23	. A . A . . . . . . . . . . . . .    
24	. T . T . . . . . . . . . . . . .    
25	. A . A . . . . . . . . . . . . .    
26	. A . A . . . . . . . . . . . . .    
27	. T . T . . . . . . . . . . . . .    
28	. A . A . . . . . . . . . . . . .    
29	. G . G . . . . . . . . . . . . .    
30	. C . C . . . . . . . . . . . . .    
31	. C . C . . . . . . . . . . . . .    
32	. C . C . . . . . . . . . . . . .    
33	. T . T . . . . . . . . . . . . .    
34	. C . C . . . . . . . . . . . . .    
35	. A . A . . . . . . . . . . . . .    
36	. A . A . . . . . . . . . . . . .    
37	. T . T . . . . . . . . . . . . .    
38	. G . G . . . . . . . . . . . . .    
39	. T . T . . . . . . . . . . . . .    
40	. T . T . . . . . . . . . . . . .    
41	. T . T . . . . . . . . . . . . .    
42	. G . G . . . . . . . . . . . . .    
43	. A . A . . . . . . . . . . . . .    
44	. A . A . . . . . . . . . . . . .    
45	. T . T . . . . . . . . . . . . .    
46	. A . A . . . . . . . . . . . . .    
47	. G . G . . . . . . . . . . . . .    
48	. A . A . . . . . . . . . . . . .    
49	. C . C . . . . . . . . . . . . .    
50	. T . T . . . . . . . . . . . . .    
51	. T . T . . . . . . . . . . . . .    
52	. A . A . . . . . . . . . . . . .    
53	. T . T . . . . . . . . . . . . .    
54	. A . A . . . . . . . . . . . . .    
55	. T . T . . . . . . . . . . . . .    
56	. C . C . . . . . . . . . . . . .    
57	. T . T . . . . . . . . . . . . .    
58	. C . C . . . . . . . . . . . . .    
59	. C . C . . . . . . . . . . . . .    
60	. A . A . . . . . . . . . . . . .    
61	. T . T . . . . . . . . . . . . .    
62	. T . T . . . . . . . . . . . . .    
63	. G . G . . . . . . . . . . . . .    
64	. T . T . . . . . . . . . . . . .    
65	. A . A . . . . . . . . . . . . .    
66	. T . T . . . . . . . . . . . . .    
67	. G . G . . . . . . . . . . . . .    
68	. T . T . . . . . . . . . . . . .    
69	. T . T . . . . . . . . . . . . .    
70	. A . A . . . . . . . . . . . . .    
71	. T . T . . . . . . . . . . . . .    
72	. C . C . . . . . . . . . . . . .    
73	. G . G . . . . . . . . . . . . .    
74	. C . C . . . . . . . . . . . . .    
75	. C . C . . . . . . . . . . . . .    
76	. T . T . . . . . . . . . . . . .    
77	. A . A . . . . . . . . . . . . .    
78	. A . A . . . . . . . . . . . . .    
79	. C . C . . . . . . . . . . . . .    
80	. T . T . . . . . . . . . . . . .    
81	. A . A . . . . . . . . . . . . .    
82	. T . T . . . . . . . . . . . . .    
83	. A . A . . . . . . . . . . . . .    
84	. A . A . . . . . . . . . . . . .    
85	. T . T . . . . . . . . . . . . .    
86	. T . T . . . . . . . . . . . . .    
87	. T . T . . . . . . . . . . . . .    
88	. G . G . . . . . . . . . . . . .    
89	. A . A . . . . . . . . . . . . .    
90	. C . C . . . . . . . . . . . . .    
91	. C . C . . . . . . . . . . . . .    
92	. T . T . . . . . . . . . . . . .    
93	. T . T . . . . . . . . . . . . .    
94	. A . A . . . . . . . . . . . . .    
95	. A . A . . . . . . . . . . . . .    
96	. A . A . . . . . . . . . . . . .    
97	. T . T . . . . . . . . . . . . .    
98	. T . T . . . . . . . . . . . . .    
99	. T . T . . . . . . . . . . . . .    
100	. A . A . . . . . . . . . . . . .    
101	. A . A . . . . . . . . . . . . .    
102	. A . A . . . . . . . . . . . . .    
103	. T . T . . . . . . . . . . . . .    
104	. C . C . . . . . . . . . . . . .    
105	. A . A . . . . . . . . . . . . .    
106	. A . A . . . . . . . . . . . . .    
107	. T . T . . . . . . . . . . . . .    
108	. C . C . . . . . . . . . . . . .    
109	. A . A . . . . . . . . . . . . .    
110	. A . A . . . . . . . . . . . . .    
111	. A . A . . . . . . . . . . . . .    
112	. T . T . . . . . . . . . . . . .    
113	. G . G . . . . . . . . . . . . .    
114	. C . C . . . . . . . . . . . . .    
115	. T . T . . . . . . . . . . . . .    
116	. T . T . . . . . . . . . . . . .    
117	. G . G . . . . . . . . . . . . .    
118	. A . A . . . . . . . . . . . . .    
119	. G . G . . . . . . . . . . . . .    
120	. A . A . . . . . . . . . . . . .    
121	. C . C . . . . . . . . . . . . .    
122	. A . A . . . . . . . . . . . . .    
123	. G . G . . . . . . . . . . . . .    
124	. A . A . . . . . . . . . . . . .    
125	. G . G . . . . . . . . . . . . .    
126	. G . G . . . . . . . . . . . . .    
127	. T . T . . . . . . . . . . . . .    
128	. A . A . . . . . . . . . . . . .    
129	. A . A . . . . . . . . . . . . .    
130	. A . A . . . . . . . . . . . . .    
131	. A . A . . . . . . . . . . . . .    
132	. A . A . . . . . . . . . . . . .    
133	. A . A . . . . . . . . . . . . .    
134	. A . A . . . . . . . . . . . . .    
135	. C . C . . . . . . . . . . . . .    
136	. A . A . . . . . . . . . . . . .    
137	. A . A . . . . . . . . . . . . .    
138	. A . A . . . . . . . . . . . . .    
139	. A . A . . . . . . . . . . . . .    
140	. A . A . . . . . . . . . . . . .    
141	. C . C . . . . . . . . . . . . .    
142	. A . A . . . . . . . . . . . . .    
143	. C . C . . . . . . . . . . . . .    
144	. A . A . . . . . . . . . . . . .    
145	. A . A . . . . . . . . . . . . .    
146	. T . T . . . . . . . . . . . . .    
147	. T . T . . . . . . . . . . . . .    
148	. C . C . . . . . . . . . . . . .    
149	. C . C . . . . . . . . . . . . .    
150	. G . G . . . . . . . . . . . . .    
151	. C . C . . . . . . . . . . . . .    
152	. A . A . . . . . . . . . . . . .    
153	. A . A . . . . . . . . . . . . .    
154	. A . A . . . . . . . . . . . . .    
155	. A . A . . . . . . . . . . . . .    
156	. A . A . . . . . . . . . . . . .    
157	. T T T . . . . . . . . . . . . .    
158	A A A A A A . A A . A A A A A A A    
159	A A A A A A . A A . A A A A A A A    
160	A A A A A A . A A . A A A A A A A    
161	A A A A A A . A A . A A A A A A A    
162	A A A A A A . A A . A A A A A A A    
163	C C C C C C . C C . C C C C C C C    
164	A A A A A A . A A . A A A A A A A    
165	A A A A A A . A A . A A A A A A A    
166	A A A A A A . A A . A A A A A A A    
167	A A A A A A . A A . A A A A A A A    
168	T T T T T T . T T . T T T T T T T    
169	A A A A A A . A A . A A A A A A A    
170	C C C C C C . C C . C C C C C C C    
171	C C C C C C . C C . C C C C C C C    
172	C C C C C C . C C . C C C C C C C    
173	A A A A A A . A A . A A A A A A A    
174	G G G G G G . G G . G G G G G G G    
175	T T T T T T . T T . T T T T T T T    
176	T T T T T T . T T . T T T T T T T    
177	T T T T T T . T T . T T T T T T T    
178	A A A A A A . A A . A A A A A A A    
179	T T T T T T . T T . T T T T T T T    
180	T T T T T T . T T . T T T T T T T    
181	C C C C C C . C C . C C C C C C C    
182	A A A A A A . A A . A A A A A A A    
183	C C C C C C . C C . C C C C C C C    
184	A A A A A A . A A . A A A A A A A    
185	A A A A A A . A A . A A A A A A A    
186	G G G G G G . G G . G G G G G G G    
187	A A A A A A . A A . A A A A A A A    
188	T T T T T T . T T . T T T T T T T    
189	T T T T T T . T T . T T T T T T T    
190	T T T T T T . T T . T T T T T T T    
191	C C C C C C . C C . C C C C C C C    
192	A A A A A A . A A . A A A A A A A    
193	C C C C C C . C C . C C C C C C C    
194	A A A A A A . A A . A A A A A A A    
195	A A A A A A . A A . A A A A A A A    
196	T T T T T T . T T . T T T T T T T    
197	T T T T T T . T T . T T T T T T T    
198	C C C C C C . C C . C C C C C C C    
199	A A A A A A . A A . A A A A A A A    
200	A A A A A A . A A . A A A A A A A    
201	A A A A A A . A A . A A A A A A A    
202	A A A A A A . A A . A A A A A A A    
203	C C C C C C . C C . C C C C C C C    
204	A A A A A A . A A . A A A A A A A    
205	T T T T T T . T T . T T T T T T T    
206	T T T T T T . T T . T T T T T T T    
207	T T T T T T . T T . T T T T T T T    
208	C C C C C C . C C . C C C C C C C    
209	C C C C C C . C C . C C C C C C C    
210	A A A A A A . A A . A A A A A A A    
211	A A A A A A . A A . A A A A A A A    
212	A A A A A A . A A . A A A A A A A    
213	T T T T T T . T T . T T T T T T T    
214	G G G G G G . G G . G G G G G G G    
215	A A A A A A . A A . A A A A A A A    
216	A A A A A A . A A . A A A A A A A    
217	A A A A A A . A A . A A A A A A A    
218	C C C C C C . C C . C C C C C C C    
219	A A A A A A . A A . A A A A A A A    
220	T T T T T T . T T . T T T T T T T    
221	C C C C C C . C C . C C C C C C C    
222	T T T T T T . T T . T T T T T T T    
223	G G G G G G . G G . G G G G G G G    
224	C C C C C C . C C . C C C C C C C    
225	C C C C C C . C C . C C C C C C C    
226	C C C C C C . C C . C C C C C C C    
227	A A A A A A . A A . A A A A A A A    
228	A A A A A A . A A . A A A A A A A    
229	A A A A A A . A A . A A A A A A A    
230	C C C C C C . C C . C C C C C C C    
231	G G G G G G . G G . G G G G G G G    
232	T T T T T T . T T . T T T T T T T    
233	A A A A A A . A A . A A A A A A A    
234	T T T T T T . T T . T T T T T T T    
235	C C C C C C . C C . C C C C C C C    
236	A A A A A A . A A . A A A A A A A    
237	A A A A A A . A A . A A A A A A A    
238	A A A A A A . A A . A A A A A A A    
239	T T T T T T . T T . T T T T T T T    
240	A A A A A A . A A . A A A A A A A    
241	C C C C C C . C C . C C C C C C C    
242	A A A A A A . A A . A A A A A A A    
243	T T T T T T . T T . T T T T T T T    
244	A A A A A A . A A . A A A A A A A    
245	C C C C C C . C C . C C C C C C C    
246	T T T T T T . T T . T T T T T T T    
247	C C C C C C . C C . C C C C C C C    
248	T T T T T T . T T . T T T T T T T    
249	A A A A A A . A A . A A A A A A A    
250	T T T T T T . T T . T T T T T T T    
251	C C C C C C . C C . C C C C C C C    
252	A A A A A A . A A . A A A A A A A    
253	A A A A A A . A A . A A A A A A A    
254	C C C C C C . C C . C C C C C C C    
255	A A A A A A . A A . A A A A A A A    
256	G G G G G G . G G . G G G G G G G    
257	G G G G G G . G G . G G G G G G G    
258	A A A A A A . A A . A A A A A A A    
259	A A A A A A . A A . A A A A A A A    
260	A A A A A A . A A . A A A A A A A    
261	A A A A A A . A A . A A A A A A A    
262	A A A A A A . A A . A A A A A A A    
263	G G G G G G . G G . G G G G G G G    
264	A A A A A A A A A A A A A A A A A    
265	A A A A A A A A A A A A A A A A A    
266	G G G G G G G G G G G G G G G G G    
267	G G G G G G G G G G G G G G G G G    
268	A A A A A A A A A A A A A A A A A    
269	A A A A A A A A A A A A A A A A A    
270	A A A A A A A A A A A A A A A A A    
271	T T T T T T T T T T T T T T T T T    
272	T T T T T T T T T T T T T T T T T    
273	A A A A A A A A A A A A A A A A A    
274	C C C C C C C C C C C C C C C C C    
275	A A A A A A A A A A A A A A A A A    
276	A A A A A A A A A A A A A A A A A    
277	A A A A A A A A A A A A A A A A A    
278	A A A A A A A A A A A A A A A A A    
279	T T T T T T T T T T T T T T T T T    
280	A A A A A A A A A A A A A A A A A    
281	A A A A A A A A A A A A A A A A A    
282	A A A A A A A A A A A A A A A A A    
283	A A A A A A A A A A A A A A A A A    
284	A A A A A A A A A A A A A A A A A    
285	A A A A A A A A A A A A A A A A A    
286	A A A A A A A A A A A A A A A A A    
287	G G G G G G G G G G G G G G G G G    
288	T T T T T T T T T T T T T T T T T    
289	G G G G G G G G G G G G G G G G G    
290	T T T T T T T T T T T T T T T T T    
291	A A A A A A A A A A A A A A A A A    
292	T - - - - T T T T T T T - - - - -  8	 indel  
293	A - - - - A A A A A A A - - - - -  8	 indel  
294	A A A A A A A A A A A A A A A A A    
295	T T T T T T T T T T T T T T T T T    
296	G G G G G G G G G G G G G G G G G    
297	T T T T T T T T T T T T T T T T T    
298	C C C C C C C C C C C C C C C C C    
299	C C C C C C C C C C C C C C C C C    
300	A A A A A A A A A A A A A A A A A    
301	T T T T T T T T T T T T T T T T T    
302	T T T T T T T T T T T T T T T T T    
303	A A A A A A A A A A A A A A A A A    
304	T T T T T T T T T T T T T T T T T    
305	T T T T T T T T T T T T T T T T T    
306	C C C C C C C C C C C C C C C C C    
307	C C C C C C C C C C C C C C C C C    
308	A A A A A A A A A A A A A A A A A    
309	C C C C C C C C C C C C C C C C C    
310	T T T T T T T T T T T T T T T T T    
311	T T T T T T T T T T T T T T T T T    
312	C C C C C C C C C C C C C C C C C    
313	T T T T T T T T T T T T T T T T T    
314	T T T T T T T T T T T T T T T T T    
315	C C C C C C C C C C C C C C C C C    
316	C C C C C C C C C C C C C C C C C    
317	A A A A A A A A A A A A A A A A A    
318	A A A A A A A A A A A A A A A A A    
319	G G G G G G G G G G G G G G G G G    
320	C C C C C C C C C C C C C C C C C    
321	C C T C C C C C C C C C T T T T T  6	 transition  
322	G G G G G G G G G G G G G G G G G    
323	A A A A A A A A A A A A A A A A A    
324	T T T T T T T T T T T T T T T T T    
325	T T T T T T T T T T T T T T T T T    
326	C C C C C C C C C C C C C C C C C    
327	T T T T T T T T T T T T T T T T T    
328	G G G G G G G G G G G G G G G G G    
329	C C C C C C C C C C C C C C C C C    
330	C C C C C C C C C C C C C C C C C    
331	A A A A A A A A A A A A A A A A A    
332	T T T T T T T T T T T T T T T T T    
333	C C C C C C C C C C C C C C C C C    
334	C C C C C C C C C C C C C C C C C    
335	C C C C C C C C C C C C C C C C C    
336	C C C C C C C C C C C C C C C C C    
337	A A A A A A A A A A A A A A A A A    
338	C C C C C C C C C C C C C C C C C    
339	C C C C C C C C C C C C C C C C C    
340	C C C C C C C C C C C C C C C C C    
341	T T T T T T T T T T T T T T T T T    
342	C C C C C C C C C C C C C C C C C    
343	A A A A A A A A A A A A A A A A A    
344	T T T T T T T T T T T T T T T T T    
345	A A A A A A A A A A A A A A A A A    
346	C C C C C C C C C C C C C C C C C    
347	T T T T T T T T T T T T T T T T T    
348	C C C C C C C C C C C C C C C C C    
349	T T T T T T T T T T T T T T T T T    
350	C C C C C C C C C C C C C C C C C    
351	T T T T T T T T T T T T T T T T T    
352	C C C C C C C C C C C C C C C C C    
353	A A A A A A A A A A A A A A A A A    
354	A A A A A A A A A A A A A A A A A    
355	G G G G G G G G G G G G G G G G G    
356	G G G G G G G G G G G G G G G G G    
357	G G G G G G G G G G G G G G G G G    
358	C C C C C C C C C C C C C C C C C    
359	C C C C C C C C C C C C C C C C C    
360	T T T T T T T T T T T T T T T T T    
361	C C C C C C C C C C C C C C C C C    
362	C C C C C C C C C C C C C C C C C    
363	A A A A A A A A A A A A A A A A A    
364	A A A A A A A A A A A A A A A A A    
365	C C C C C C C C C C C C C C C C C    
366	A A A A A A A A A A A A A A A A A    
367	T T T T T T T T T T T T T T T T T    
368	G G G G G G G G G G G G G G G G G    
369	A A A A A A A A A A A A A A A A A    
370	T T T T T T T T T T T T T T T T T    
371	G G G G G G G G G G G G G G G G G    
372	A A A A A A A A A A A A A A A A A    
373	T T T T T T T T T T T T T T T T T    
374	G G G G G G G G G G G G G G G G G    
375	C C C C C C C C C C C C C C C C C    
376	T T T T T T T T T T T T T T T T T    
377	G G G G G G G G G G G G G G G G G    
378	T T T T T T T T T T T T T T T T T    
379	T T T T T T T T T T T T T T T T T    
380	T T T T T T T T T T T T T T T T T    
381	C C C C C C C C C C C C C C C C C    
382	C C C C C C C C C C C C C C C C C    
383	T T T T T T T T T T T T T T T T T    
384	C C C C C C C C C C C C C C C C C    
385	T T T T T T T T T T T T T T T T T    
386	G G G G G G G G G G G G G G G G G    
387	A A A A A A A A A A A A A A A A A    
388	T T T T T T T T T T T T T T T T T    
389	T T T T T T T T T T T T T T T T T    
390	A A A A A A A A A A A A A A A A A    
391	C C C C C C C C C C C C C C C C C    
392	C C C C C C C C C C C C C C C C C    
393	A A A A A A A A A A A A A A A A A    
394	C C C C C C C C C C C C C C C C C    
395	C C C C C C C C C C C C C C C C C    
396	A A A A A A A A A A A A A A A A A    
397	T T T T T T T T T T T T T T T T T    
398	G G G G G G G G G G G G G G G G G    
399	C C C C C C C C C C C C C C C C C    
400	C C C C C C C C C C C C C C C C C    
401	T T T T T T T T T T T T T T T T T    
402	A A A A A A A A A A A A A A A A A    
403	T T T T T T T T T T T T T T T T T    
404	C C C C C C C C C C C C C C C C C    
405	G G G G G G G G G G G G G G G G G    
406	T T T T T T T T T T T T T T T T T    
407	G G G G G G G G G G G G G G G G G    
408	T T T T T T T T T T T T T T T T T    
409	T T T T T T T T T T T T T T T T T    
410	C C C C C C C C C C C C C C C C C    
411	T T T T T T T T T T T T T T T T T    
412	G G G G G G G G G G G G G G G G G    
413	T T T T T T T T T T T T T T T T T    
414	A A A A A A A A A A A A A A A A A    
415	T T T T T T T T T T T T T T T T T    
416	T T T T T T T T T T T T T T T T T    
417	C C C C C C C C C C C C C C C C C    
418	C C C C C C C C C C C C C C C C C    
419	C C C C C C C C C C C C C C C C C    
420	C C C C C C C C C C C C C C C C C    
421	C C C C C C C C C C C C C C C C C    
422	A A A A A A A A A A A A A A A A A    
423	G G G G G G G G G G G G G G G G G    
424	G G G G G G G G G G G G G G G G G    
425	G G G G G G G G G G G G G G G G G    
426	G G G G G G G G G G G G G G G G G    
427	C C C C C C C C C C C C C C C C C    
428	C C C C C C C C C C C C C C C C C    
429	A A A A A A A A A A A A A A A A A    
430	T T T T T T T T T T T T T T T T T    
431	C C C C C C C C C C C C C C C C C    
432	T T T T T T T T T T T T T T T T T    
433	C C C C C C C C C C C C C C C C C    
434	C C C C C C C C C C C C C C C C C    
435	A A A A A A A A A A A A A A A A A    
436	A A A A A A A A A A A A A A A A A    
437	G G G G G G G G G G G G G G G G G    
438	C C C C C C C C C C C C C C C C C    
439	A A A A A A A A A A A A A A A A A    
440	G G G G G G G G G G G G G G G G G    
441	T T T T T T T T T T T T T T T T T    
442	C C C C C C C C C C C C C C C C C    
443	A A A A A A A A A A A A A A A A A    
444	T T T T T T T T T T T T T T T T T    
445	C C C C C C C C C C C C C C C C C    
446	C C C C C C C C C C C C C C C C C    
447	A A A A A A A A A A A A A A A A A    
448	T T T T T T T T T T T T T T T T T    
449	C C C C C C C C C C C C C C C C C    
450	A A A A A A A A A A A A A A A A A    
451	C C C C C C C C C C C C C C C C C    
452	C C C C C C C C C C C C C C C C C    
453	A A A A A A A A A A A A A A A A A    
454	A A A A A A A A A A A A A A A A A    
455	G G G G G G G G G G G G G G G G G    
456	T T T T T T T T T T T T T T T T T    
457	T T T T T T T T T T T T T T T T T    
458	C C C C C C C C C C C C C C C C C    
459	A A A A A A A A A A A A A A A A A    
460	T T T T T T T T T T T T T T T T T    
461	G G G G G G G G G G G G G G G G G    
462	A A A A A A A A A A A A A A A A A    
463	A A A A A A A A A A A A A A A A A    
464	G G G G G G G G G G G G G G G G G    
465	G G G G G G G G G G G G G G G G G    
466	G G G G G G G G G G G G G G G G G    
467	G G G G G G G G G G G G G G G G G    
468	T T T T T T T T T T T T T T T T T    
469	C C C C C C C C C C C C C C C C C    
470	A A A A A A A A A A A A A A A A A    
471	A A A A A A A A A A A A A A A A A    
472	A A A A A A A A A A A A A A A A A    
473	T T T T T T T T T T T T T T T T T    
474	C C C C C C C C C C C C C C C C C    
475	C C C C C C C C C C C C C C C C C    
476	T T T T T T T T T T T T T T T T T    
477	. C C C C C C C C C C C C C C C C    
478	. G G G G G G G G G G G G G G G G    
479	. C C C C C C C C C C C C C C C C    
480	. A A A A A A A A A A A A A A A A    
481	. G G G G G G G G G G G G G G G G    
482	. G G G G G G G G G G G G G G G G    
483	. A A A A A A A A A A A A A A A A    
484	. C C C C C C C C C C C C C C C C    
485	. T T T T T T T T T T T T T T T T    
486	. C C C C C C C C C C C C C C C C    
487	. C C C C C C C C C C C C C C C C    
488	. T T T T T T T T T T T T T T T T    
489	. T T T T T T T T T T T T T T T T    
490	. G G G G G G G G G G G G G G G G    
491	. G G G G G G G G G G G G G G G G    
492	. A A A A A A A A A A A A A A A A    
493	. C C C C C C C C C C C C C C C C    
494	. C C C C C C C C C C C C C C C C    
495	. T T T T T T T T T T T T T T T T    
496	. G G G G G G G G G G G G G G G G    
497	. C C C C C C C C C C C C C C C C    
498	. C C C C C C C C C C C C C C C C    
499	. T T T T T T T T T T T T T T T T    
500	. G G G G G G G G G G G G G G G G    
501	. C C C C C C C C C C C C C C C C    
502	. C C C C C C C C C C C C C C C C    
503	. A A A A A A A A A A A A A A A A    
504	. C C C C C C C C C C C C C C C C    
505	. C C C C C C C C C C C C C C C C    
506	. G G G G G G G G G G G G G G G G    
507	. T T T T T T T T T T T T T T T T    
508	. T T T T T T T T T T T T T T T T    
509	. C C C C C C C C C C C C C C C C    
510	. A A A A A A A A A A A A A A A A    
511	. G G G G G G G G G G G G G G G G    
512	. C C C C C C C C C C C C C C C C    
513	. T T T T T T T T T T T T T T T T    
514	. T T T T T T T T T T T T T T T T    
515	. C C C C C C C C C C C C C C C C    
516	. A A A A A A A A A A A A A A A A    
517	. G G G G G G G G G G G G G G G G    
518	. A A A A A A A A A A A A A A A A    
519	. G G G G G G G G G G G G G G G G    
520	. A A A A A A A A A A A A A A A A    
521	. A A A A A A A A A A A A A A A A    
522	. A A A A A A A A A A A A A A A A    
523	. G G G G G G G G G G G G G G G G    
524	. C C C C C C C C C C C C C C C C    
525	. T T T T T T T T T T T T T T T T    
526	. T T T T T T T T T T T T T T T T    
527	. C C C C C C C C C C C C C C C C    
528	. T T T T T T T T T T T T T T T T    
529	. T T T T T T T T T T T T T T T T    
530	. G G G G G G G G G G G G G G G G    
531	. T T T T T T T T T T T T T T T T    
532	. C C C C C C C C C C C C C C C C    
533	. C C C C C C C C C C C C C C C C    
534	. A A A A A A A A A A A A A A A A    
535	. T T T T T T T T T T T T T T T T    
536	. G G G G G G G G G G G G G G G G    
537	. A A A A A A A A A A A A A A A A    
538	. A A A A A A A A A A A A A A A A    
539	. C C C C C C C C C C C C C C C C    
540	. T T T T T T T T T T T T T T T T    
541	. T T T T T T T T T T T T T T T T    
542	. T T T T T T T T T T T T T T T T    
543	. T T T T T T T T T T T T T T T T    
544	. T T T T T T T T T T T T T T T T    
545	. C C C C C C C C C C C C C C C C    
546	. A A A A A A A A A A A A A A A A    
547	. A A A A A A A A A A A A A A A A    
548	. C C C C C C C C C C C C C C C C    
549	. T T T T T T T T T T T T T T T T    
550	. C C C C C C C C C C C C C C C C    
551	. T T T T T T T T T T T T T T T T    
552	. G G G G G G G G G G G G G G G G    
553	. G G G G G G G G G G G G G G G G    
554	. T T T T T T T T T T T T T T T T    
555	. G G G G G G G G G G G G G G G G    
556	. G G G G G G G G G G G G G G G G    
557	. G G G G G G G G G G G G G G G G    
558	. T T T T T T T T T T T T T T T T    
559	. G G G G G G G G G G G G G G G G    
560	. T T T T T T T T T T T T T T T T    
561	. G G G G G G G G G G G G G G G G    
562	. C C C C C C C C C C C C C C C C    
563	. T T T T T T T T T T T T T T T T    
564	. T T T T T T T T T T T T T T T T    
565	. T T T T T T T T T T T T T T T T    
566	. A A A A A A A A A A A A A A A A    
567	. C C C C C C C C C C C C C C C C    
568	. T T T T T T T T T T T T T T T T    
569	. C C C C C C C C C C C C C C C C    
570	. A A A A A A A A A A A A A A A A    
571	. T T T T T T T T T T T T T T T T    
572	. G G G G G G G G G G G G G G G G    
573	. T T T T T T T T T T T T T T T T    
574	. T T T T T T T T T T T T T T T T    
575	. T T T T T T T T T T T T T T T T    
576	. C C C C C C C C C C C C C C C C    
577	. T T T T T T T T T T T T T T T T    
578	. T T T T T T T T T T T T T T T T    
579	. T T T T T T T T T T T T T T T T    
580	. T T T T T T T T T T T T T T T T    
581	. T T T T T T T T T T T T T T T T    
582	. G G G G G G G G G G G G G G G G    
583	. T T T T T T T T T T T T T T T T    
584	. A A A A A A A A A A A A A A A A    
585	. G G G G G G G G G G G G G G G G    
586	. T T T T T T T T T T T T T T T T    
587	. G G G G G G G G G G G G G G G G    
588	. G G G G G G G G G G G G G G G G    
589	. G G G G G G G G G G G G G G G G    
590	. C C C C C C C C C C C C C C C C    
591	. C C C C C C C C C C C C C C C C    
592	. T T T T T T T T T T T T T T T T    
593	. T T T T T T T T T T T T T T T T    
594	. C C C C C C C C C C C C C C C C    
595	. A A A A A A A A A A A A A A A A    
596	. G G G G G G G G G G G G G G G G    
597	. T T T T T T T T T T T T T T T T    
598	. T T T T T T T T T T T T T T T T    
599	. C C C C C C C C C C C C C C C C    
600	. G G G G G G G G G G G G G G G G    
601	. T T T T T T T T T T T T T T T T    
602	. G G G G G G G G G G G G G G G G    
603	. A A A A A A A A A A A A A A A A    
604	. G G G G G G G G G G G G G G G G    
605	. A A A A A A A A A A A A A A A A    
606	. C C C C C C C C C C C C C C C C    
607	. T T T T T T T T T T T T T T T T    
608	. T T T T T T T T T T T T T T T T    
609	. G G G G G G G G G G G G G G G G    
610	. C C C C C C C C C C C C C C C C    
611	. G C G G G . G G G G G G G G G G    
612	. T . T T T . T T T T T T C T T T    
613	. T . T T T . T T T T T T . T T T    
614	. T . T T T . T T T T T T . T T T    
615	. T . T G G . G G G G G G . G G G    
616	. . . . T T . T T G T T T . T T T    
617	. . . . G G . . G . G G G . G G G    
618	. . . . A A . . A . A A A . A A A    
619	. . . . A A . . A . A A A . A A A    
620	. . . . A A . . A . A A A . A A A    
621	. . . . A A . . A . A A A . A A A    
622	. . . . A A . . A . A A A . A A A    
623	. . . . G G . . G . G G G . G G G    
624	. . . . C C . . C . C C C . C C C    
625	. . . . G G . . G . G G G . G G G    
626	. . . . G G . . G . G G G . G G G    
627	. . . . A A . . A . A A A . C C A    
628	. . . . A A . . A . A A A . . . A    
629	. . . . A A . . A . A A A . . . A    
630	. . . . G G . . G . G G G . . . G    
631	. . . . C C . . C . C C C . . . C    
632	. . . . A A . . C . C A A . . . A    
633	. . . . T T . . . . . T T . . . T    
634	. . . . G C . . . . . C G . . . G    
635	. . . . C . . . . . . . C . . . C    
636	. . . . G . . . . . . . G . . . G    
637	. . . . C . . . . . . . C . . . C    
638	. . . . C . . . . . . . A . . . A    
639	. . . . . . . . . . . . A . . . A    
640	. . . . . . . . . . . . C . . . C    
641	. . . . . . . . . . . . G . . . G    
642	. . . . . . . . . . . . A . . . A    
643	. . . . . . . . . . . . G . . . G    
644	. . . . . . . . . . . . C . . . C    
645	. . . . . . . . . . . . A . . . A    
646	. . . . . . . . . . . . G . . . G    
647	. . . . . . . . . . . . A . . . A    
648	. . . . . . . . . . . . G . . . G    
649	. . . . . . . . . . . . C . . . C    
650	. . . . . . . . . . . . C . . . C    
651	. . . . . . . . . . . . C . . . C    
652	. . . . . . . . . . . . C . . . C    
653	. . . . . . . . . . . . C . . . C    
654	. . . . . . . . . . . . C . . . C    
655	. . . . . . . . . . . . C . . . C
```
